# Site-Specific Raman Probes Reveal Droplet Aging and Residue-Level Fibril Polymorphism in TDP-43_CTD_

**DOI:** 10.1021/jacs.6c07727

**Published:** 2026-06-02

**Authors:** Matthew D. Watson, Jennifer C. Lee

**Affiliations:** Laboratory of Protein Conformation and Dynamics, Biochemistry and Biophysics Center, National Heart, Lung, and Blood Institute, 2511 National Institutes of Health, Bethesda, Maryland 20892, United States

## Abstract

The C-terminal domain of TAR DNA-binding protein 43 (TDP-43_CTD_) drives both liquid–liquid phase separation (LLPS) and amyloid formation. Understanding how TDP-43_CTD_ droplets convert into amyloid aggregates, a process implicated in amyotrophic lateral sclerosis and frontotemporal dementia, requires methodology capable of site-specific structural characterization with spatial resolution. Here, we used confocal Raman spectroscopy in conjunction with an alkyne-modified amino acid (4-ethynyl-l-phenylalanine, F_CC_) to probe aging in individual TDP-43_CTD_ droplets at seven aromatic sites. While nascent droplets are composed of disordered proteins, β-sheet conformers develop in aged droplets and amyloid aggregates. All three states are spectrally distinct *via* the alkyne stretching band, with sensitivity that varies depending on the aromatic site probed. C-terminal sites (Y374F_CC_, W385F_CC_, and F397F_CC_) are highly sensitive amyloid probes, revealing multiple polymorphs at the single-residue level that are not resolvable by global secondary structure or morphological characterization alone. Strikingly, while W334F_CC_ abolishes β-sheet formation in droplets, *de novo* aggregation still occurs, demonstrating that droplet aging is not required for amyloid formation. Given its broad applicability to other proteins and compatibility with cellular imaging, this work establishes a generalizable approach for investigating conformational changes underlying LLPS and amyloid formation *in cellulo*.

## Graphical abstract

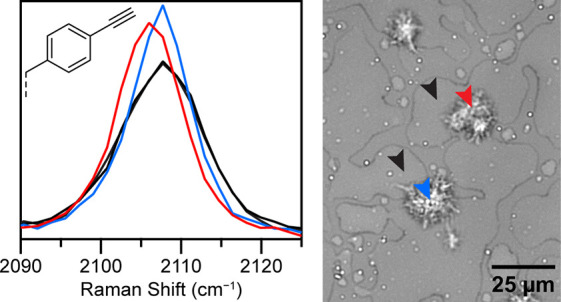

Amyloid fibrils composed of the C-terminal domain of TAR DNA-binding protein 43 (TDP-43_CTD_) are deposited in tissues of amyotrophic lateral sclerosis (ALS) and frontotemporal dementia patients.^1,2^ Aberrant TDP-43_CTD_ aggregation is proposed to occur in stress granules, biomolecular condensates formed *via* liquid–liquid phase separation (LLPS) induced by cellular stress.^3^ TDP-43_CTD_ (residues 274–414, Figure 1A) has a strong disease-connection, harboring most ALS-related mutations^4^ and playing a critical role in both LLPS and amyloid formation.^5^ Here, we investigated the relationship between TDP-43_CTD_ droplet aging, a liquid-to-solid transition,^6^ and the development of β-sheet-rich amyloid structure *in vitro* by confocal Raman spectroscopy. Vibrational spectroscopy has been demonstrated to be a particularly powerful tool for studying structural changes in phase-separating systems.^7–10^ By coupling Raman spectroscopic probes with the spatial resolution of a confocal microscope, we selectively interrogated individual aged droplets *vs*. amyloid aggregates, examining global secondary structural (amide-I and -III bands^11,12^ ) and local chemical environmental (alkyne stretch of 4-ethynyl-l-phenylalanine (F_CC_)^13^) differences that are otherwise indistinguishable by bulk measurements.

**Figure 1:**
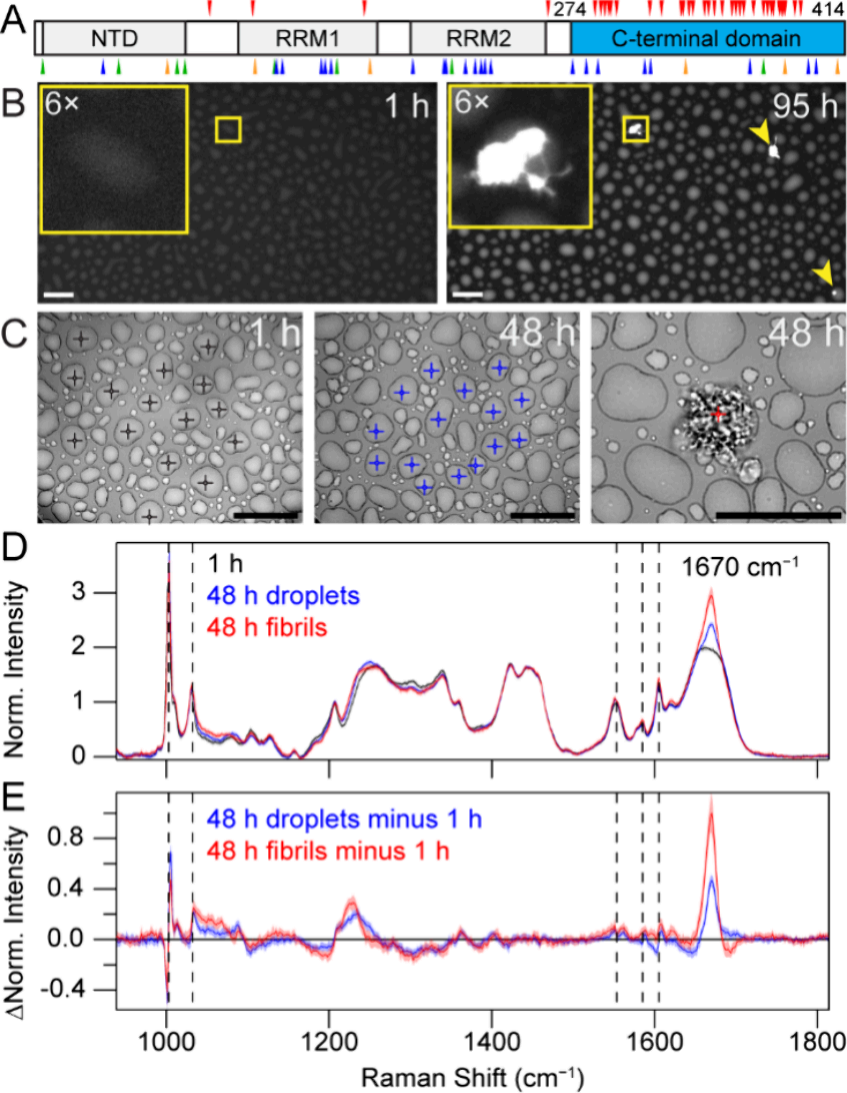
TDP-43_CTD_ droplet aging and amyloid formation. (A) TDP-43 schematic showing N- (NTD) and C-terminal domains, RNA recognition motifs (RRM), and ALS-mutations (red arrows). Phe (blue), Tyr (green), and Trp (orange) sites are indicated by arrows. (B) Thioflavin-T fluorescence images of TDP-43_CTD_ droplets showing the same field of view after 1 h (left) and 95 h (right). Arrows indicate fibrils. Scale bars are 100 μm. Insets: magnified areas. (C) Bright-field images of TDP-43_CTD_ droplets after 1 h and 48 h. Acquisition positions of nascent droplet (black), aged droplet (blue), and fibril (red) spectra are indicated. A representative image of fibrils is shown. Scale bars are 50 μm. (D) Raman spectra (normalized to the C–H deformation area) of nascent droplets (black), aged droplets (blue), and fibrils (red) shown in panel C. Averages (lines) and standard deviations (shaded) are shown (*n* = 16). Dashed lines indicate aromatic residue peaks. (E) Difference spectra of aged droplets (blue) and fibrils (red) from nascent droplets shown in panel D.

TDP-43_CTD_ droplet aging was surveyed by time-lapse fluorescence imaging using thioflavin-T (ThT) as an extrinsic probe of amyloid structure.^14^ Upon aging, all droplets exhibit moderate ThT fluorescence increases with a few intensely fluorescent aggregates (herein referred to as fibrils, Figure 1B arrows). The rarity of fibrils is difficult to reconcile with any aggregation model that places LLPS on pathway to amyloid formation (representative transmission electron micrograph is shown in Supporting Information Figure S1). Instead, this suggests that changes in the majority of droplets do not produce fibrils (Figure 1C), which is supported by Raman spectra of individual droplets and fibrils at 48 h, where distinct spectral features are observed (Figure 1D). Nascent droplets (1 h) have a broad amide-I band (Figure 1D, black) compared to both aged droplets (Figure 1D, blue) and fibrils (Figure 1D, red), indicating that TDP-43_CTD_ is largely disordered in the fluid state as previously reported.^12^ Difference spectra produced by subtracting the average nascent droplet spectrum show a greater amide-I band intensity increase and peak narrowing at 1670 cm^–1^ for fibrils (Figure 1E, red) compared to aged droplets (Figure 1E, blue), indicating higher β-sheet content and structural order in the amyloid state. Likewise, the amide-III band is narrower for fibrils, pointing to a more-ordered ensemble of structures. Beyond secondary structure, both forms exhibit changes in aromatic residue bands (Figure 1E, dashed lines), however site-specific analysis is prohibited by the multiplicity of these residues (Figure 1A).

To probe individual aromatic residues, we introduced a unique Raman signal by making single-point mutations with the unnatural amino acid (UAA) F_CC_, a minimally perturbative substitution (Figure 2A), *via* amber codon suppression using an evolved tRNA synthetase/tRNA pair.^15^ Thus far, the coupled use of UAAs and vibrational spectroscopy in amyloidogenic proteins is quite limited with one study demonstrating the intracellular localization of F_CC_-containing α-synuclein fibrils^13^ and a few reports on amylin^16^ and Aβ peptides using 4-cyano-l-phenylalanine.^17,18^ The alkyne stretching band of F_CC_ (C≡C) is environmentally sensitive and resolved from other protein peaks.^13^ In the seven F_CC_-variants studied (Figure 2B), the C≡C frequency (ν_max_ = 2107 cm^–1^) and width (fwhm = 12 cm^–1^) in nascent droplets were identical with small but consistent intensity differences for sites 334, 385, and 397 (Figure 2C). Thus, their local chemical environments appear similar, though nuances are apparent even in the disordered conformational state of nascent droplets. The C≡C band is slightly red-shifted and broadened in nascent droplets relative to the model compound, N-acetyl-4-ethynyl-l-phenylalanine methyl ester in water (ν_max_ = 2109 cm^–1^, fwhm = 9.4 cm^–1^ (Figure S2)), consistent with chemical environmental differences within protein condensates compared to that of bulk water. Upon incubation, the C≡C band undergoes distinct, site-specific changes for both aged droplets and fibrils (Figure 2D–K). While the only large intensity change occurs in W385F_CC_ aged droplets (Figure 2J, blue), all sites except F367F_CC_ show spectral sensitivity to aging.

**Figure 2:**
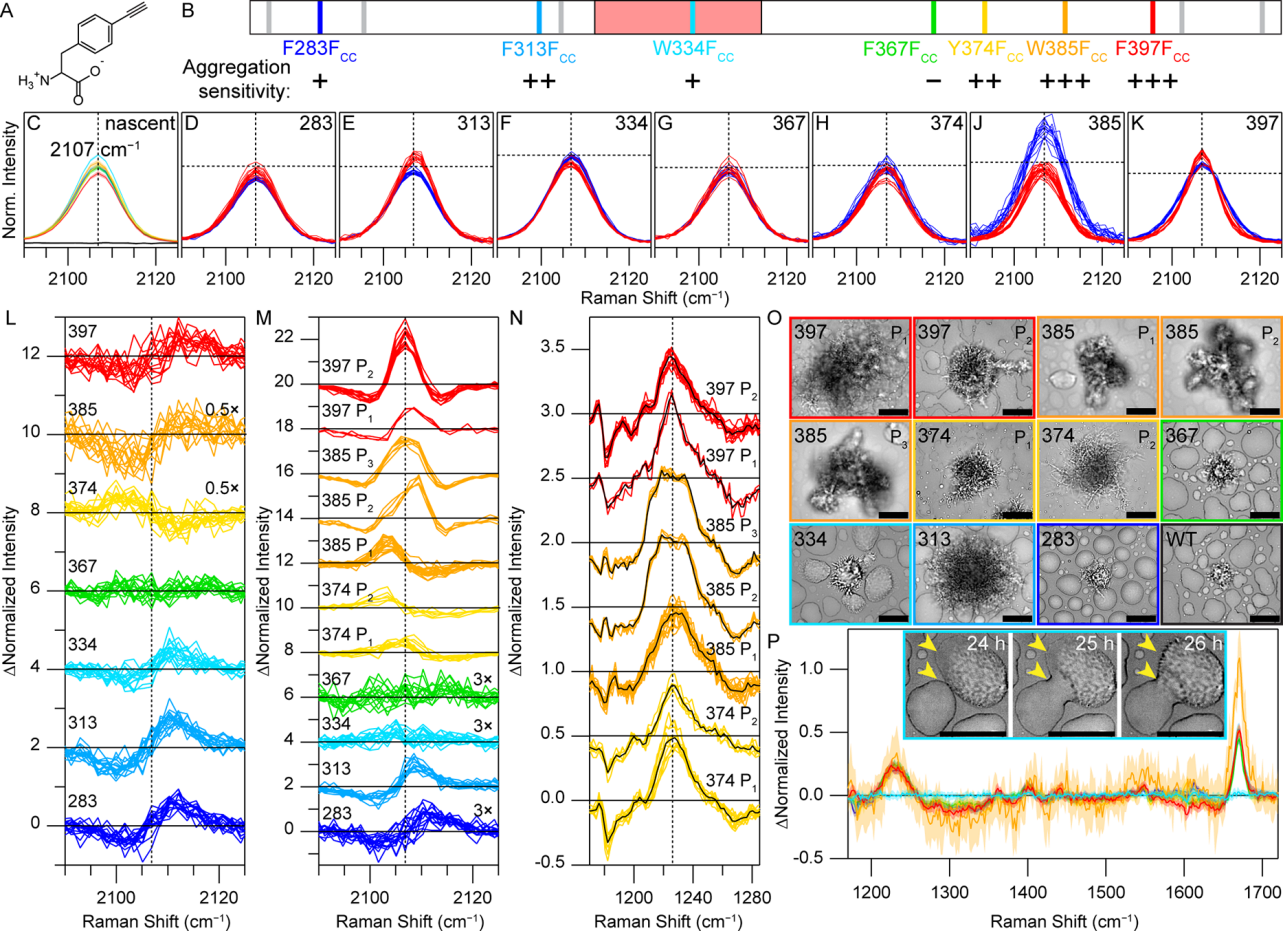
F_CC_, a site-specific conformational Raman probe. (A) Structure of F_CC_. (B) TDP-43_CTD_ schematic showing F_CC_ sites in this study (colored), other aromatic sites (gray), and the conserved region^5^ (pink). Bottom: C≡C fibrillization sensitivity ranking. (C) C≡C of nascent droplets (normalized to the C–H deformation area). Average (line, *n* = 16) and standard deviations (shaded) are shown. Colors correspond to panel B. The black line shows the wild-type protein spectrum from Figure 1D. (D–K) C≡C of aged droplets (blue) and fibrils (red) (normalized to the C–H deformation area). Peak position and intensity (dotted line) for respective nascent droplets are shown. F_CC_ positions as labeled. Full spectra are shown in Figures S3–S9. Individual C≡C difference spectra of (L) aged droplets and (M) fibrils minus nascent droplets (normalized to the C≡C area). Spectrally distinct polymorphs (P) are grouped as P1–P3. Dashed lines indicate peak position in nascent droplets. Colors correspond to panel B. Full spectra are shown in Figures S10 and S11. (N) Amide-III difference spectra of Y374F_CC_ (yellow), W385F_CC_ (orange), and F397F_CC_ (red) fibril polymorphs minus nascent droplets (normalized to the C–H deformation area). Individual and average (black) spectra are shown. Polymorphs are labeled as in panel M. Full spectra are shown in Figure S12. (O) Representative bright-field images of fibrils at 48 h for each mutant as labeled. Polymorphs are labeled as in panel M. Scale bars are 25 μm. (P) Difference spectra of aged droplets minus nascent droplets spanning amide-I and -III bands (normalized to the C–H deformation area). Colors correspond to panel B. Average (line, *n* = 16) and standard deviation (shaded) are shown. Insets: Bright-field images of W334F_CC_ from 24 to 26 h showing droplet fluidity (arrows). Scale bars are 25 μm.

To evaluate C≡C shifts which report on environmental changes due to local electronic interactions with water and nearby chemical moieties,^19^ spectra were normalized to the C≡C area, and difference spectra against the respective nascent droplets were calculated. Highly consistent changes were seen across all aged droplets for each mutant with site-specific differences (Figure 2L). All sites exhibited blue-shifts except for Y374F_CC_ (red shift) and F367F_CC_ (no change). Relative water exposure leads to varying F_CC_ shifts, where a red shift can be associated with side chain protection as seen in the fibrillar state of α-synuclein.^13^ Although C≡C solvatochromism is reasonably well predicted from a combination of solvent parameters (Figure S2), interpreting these frequency shifts in terms of specific biomolecular interactions is not straightforward. However, recent computational work is beginning to elucidate the underlying mechanisms that determine C≡C shift and shape.^20^ Nonetheless, the C≡C data reveal that the environment occupied by aromatic residues changes as fluid droplets age, and given the low droplet-to-droplet spectral variation within each mutant, there appears to be a broadly similar ensemble of structures in each aged droplet.

Remarkably, fibrils show both C≡C variations within each mutant and between mutants (Figure 2M). Whereas some residues (F283F_CC_, F313F_CC_, and F367F_CC_) are similar in aged droplets and fibrils, others differ. Interestingly, W334F_CC_ remains unchanged in the fibrillar state, whereas Y374F_CC_, W385F_CC_, and F397F_CC_ are particularly sensitive, revealing the presence of multiple populations or polymorphs (P). This is intriguing because W334F_CC_ is expected to be conformationally sensitive as it lies within the conserved region (Figure 2B) deemed essential for LLPS and oligomerization,^21^ yet our data demonstrate that C-terminal aromatic interactions are substantially more impacted during amyloid formation, pointing to their roles in aggregation. This is broadly in agreement with TDP-43_CTD_ multimer models that show formation of intermolecular contacts by aromatic residues.^22^ Vibrational spectroscopy has previously demonstrated sensitivity to polymorphism, with two-dimensional infrared spectroscopy used in conjunction with isotopically labeled backbone carbonyls to detect distinct amyloid structures of amylin.^23,24^ However, the data presented here is, to our knowledge, the first demonstration of spatially resolved detection of amyloid polymorphism by a side-chain probe.

Within some polymorphs, C≡C narrowing is observed (*i.e.*, W385F_CC_ P_2_, W385F_CC_ P_3_, and F397F_CC_ P_2_), indicating that these residues adopt highly ordered conformers, especially in F397F_CC_ P_2_ (fwhm = 9 cm^–1^). For comparison, the C≡C widths of all other TDP-43_CTD_ sites (fwhm ∼ 12 cm^–1^) are comparable to F_CC_ labels in the α-synuclein fibril core.^13^ Using these F_CC_-based classifications, spectral differences are now discernible in the amide-III region (Figure 2N), corroborating that the polymorphs indeed adopt distinct conformations. We note that amide-III band shape analysis alone was sufficient to detect polymorphs only for W385F_CC_, revealing that TDP-43_CTD_ amyloid formation is particularly sensitive to mutation at position 385, identifying this as a key residue in this process. Interestingly, W385G is an ALS-mutation (Figure 1A), supporting the importance of this residue in TDP-43 behavior. Notwithstanding, none of these polymorphs are distinguishable by microscopic morphology (Figure 2O), reaffirming the benefit of Raman spectroscopy for *in situ* structural analysis.

A striking observation was the abolishment of β-sheet development in W334F_CC_ droplets, apparent from the absence of changes in the amide-I and -III bands (Figure 2P, cyan). Droplets remain fluid as evidenced by observations of fusion events after 24 h (Figure 2P insets). Despite this, W334F_CC_ forms fibrils (Figure 2O), showing that droplet aging can be decoupled from amyloid formation.^25^ This shows that β-sheet structures formed upon droplet aging do not necessarily represent fibrillar intermediates. Interestingly, the C≡C band of W334F_CC_ droplets blue shifts similarly to other sites over 48 h (Figure 2L), revealing that changes in F_CC_ contacts and/or local polarity occur in droplets even in the absence of β-sheet development. This expands on previous work^26^ that identified Trp residues as key players in both LLPS and fibril formation.

Collectively, our work establishes that spatially resolved F_CC_ Raman spectra reveal conformational changes during TDP-43_CTD_ droplet aging with site-dependent sensitivity (Figure 2B, bottom) that are not detectable in bulk ensemble measurements. C≡C analysis demonstrates the coexistence of multiple amyloid polymorphs with unique side-chain conformers near the C-terminus. This may have clinical relevance in light of the connection between amyloid polymorphism and disease phenotype.^1,2^ Finally, the unique behavior of W334F_CC_ provided new mechanistic insights, demonstrating that β-sheet development within droplets is not necessary for amyloid formation, challenging a common hypothesis that droplets serve as nucleation sites promoting fibril formation. Looking forward, the incorporation of C≡C probes into endogenous TDP-43 has the potential to answer whether fibril formation occurs in cytosolic stress granules, where amide band analysis alone is difficult.

## Supplementary Material



## Data Availability

All data have been deposited in Figshare at https://doi.org/10.25444/nhlbi.31298425.

## Notes

Materials and methods, TEM image of fibrils, solvent series data for N-acetyl-4-ethynyl-l-phenylalanine methyl ester, and full Raman spectra and difference spectra (PDF)

## Abbreviations

ALS: amyotrophic lateral sclerosis
C≡C: alkyne stretching vibrational band
F_CC_: 4-ethynyl-l-phenylalanine
fwhm: full width at half-maximum.
LLPS: liquid–liquid phase separation
TDP-43: transactive response DNA-binding protein 43
TDP-43_CTD_: C-terminal domain of transactive response DNA-binding protein 43
ThT: thioflavin T
UAA: unnatural amino acid
ν_max_: Raman shift frequency maximum
