# Supplementary material for: Site-Specific Raman Probes Reveal Droplet Aging and Residue-Level Fibril Polymorphism in TDP-43CTD
Source: J Am Chem Soc. 2026 Jun 2;148(23):23494–8. doi: 10.1021/jacs.6c07727 (PMC13281513; doi:10.1021/jacs.6c07727)
Supplement: Supplementary file 1 [file ja6c07727_si_001.pdf]

Supporting Information for

Site-specific Raman Probes Reveal Droplet Aging and Residue-level Fibril Polymorphism in TDP-43$_{CTD}$

Matthew D. Watson and Jennifer C. Lee*

Laboratory of Protein Conformation and Dynamics, Biochemistry and Biophysics Center, National Heart, Lung, and Blood Institute, National Institutes of Health, Bethesda, MD 20892

Materials and Methods

Reagents. Chemicals were obtained from Sigma unless otherwise noted.

Protein expression and purification. TDP-43$_{CTD}$ was expressed in *E. coli* BL21(DE3) (New England BioLabs) transformed with pET21a(+)-Thio$_6$-His$_6$-TEV-TARDBP(Δ1-273) (GenScript) following established protocol.[1] For F$_{CC}$ incorporation, an amber (tag) stop codon was introduced at the target site in TARDBP(Δ1–273) and cells were co-transformed with pDule2-pCNF (Addgene plasmid # 85495).[2] Plasmids for F313tag, W334tag and Y374tag were purchased from GenScript whereas F283tag, F367tag, W385tag, and F397tag were made by standard site-directed mutagenesis using the primers listed below. Constructs were verified by DNA sequencing (Psomagen, USA).

F283tag_for: 5' GTGGTAATCCGGGTGGTTAGGGCAACCAGGGTG 3'
F283tag_rev: 5' CACCCTGGTTGCCCTAACCACCCGGATTACCAC 3'
F367tag_for: 5' CAGCGTGAACCGAATCAAGCGTAGGGTAGCGGTAACA 3'
F367tag_rev: 5' TGTTACCGCTACCCTACGCTTGATTCGGTTCACGCTG 3'
W385tag_for: 5' GTGCGGCAATCGGCTAGGGTAGCGCAA 3'
W385tag_rev: 5' TTGCGCTACCCTAGCCGATTGCCGCAC 3'
F397tag_for: 5' CGGGCAGCGGTAGCGGTTAGAACGGCGGCT 3'
F397tag_rev: 5' AGCCGCCGTTCTAACCGCTACCGCTGCCCG 3'

Glycerol stocks were prepared in LB media and used to inoculate starter cultures in media adapted from Hammill *et al.*[3] (KH_2PO_4 13 g/L, K_2HPO_4 10 g/L, Na_2HPO_4 9 g/L, K_2SO_4 2.4 g/L, NH_4Cl 0.1% w/w, $MgSO_4$ 5 mM, glucose 0.24% w/w, and trace elements) under selective pressure of carbenicillin (50 mg/L) and spectinomycin (60 mg/L). Expression cultures were grown in identical media supplemented with F$_{CC}$ (30 mg/L, MedChem Express) at 37 °C to an optical density of 0.5 at 600 nm, the temperature was reduced to 18 °C, and protein expression was induced by addition of IPTG to a final concentration of 1 mM. Cells were harvested after 16 h and stored at −80 °C until purification.

Cells were thawed and resuspended in Tris buffer (40 mM Tris, 500 mM NaCl, 1 mM ethylenediaminetetraacetic acid (EDTA), 1 mM phenylmethanesulfonyl fluoride (PMSF), pH 8.0) and lysed by sonication on ice (20 min, 50% duty cycle, amplitude 5, Branson Sonifier 450). Cellular debris and inclusion bodies were pelleted by centrifugation at 24,000 rcf at 4 °C, and the supernatant discarded. Thio$_6$-His$_6$-TEV-TDP-43$_{CTD}$ was resolubilized from inclusion bodies by

slow rotary mixing at 4 °C for 16 h in denaturing buffer (7 M urea [Thermo Scientific J75826-A7], 20 mM Tris, 500 mM NaCl, 20 mM imidazole [Thermo Scientific 396745000], 1 mM PMSF, pH 8.0). Cellular debris was pelleted by centrifugation at 24,000 rcf, and the supernatant was pumped onto a HisPrep 16/10 FF column (Cytiva) and eluted with an imidazole gradient. Protein was desalted into TEV cleavage buffer (1 M guanidine HCl [Thermo Scientific 15502-016], 20 mM Tris, pH 8.0) using a HiPrep 26/10 desalting column (Cytiva). Protein concentration was estimated from the absorbance at 280 nm based on amino acid content, 1,2-dithiothreitol was added to a final concentration of 1 mM, and TEV protease (GenScript) was added at a ratio of 3.5 U per nmol of $Thio_6$-His_6-TEV-TDP-43_{CTD}. The cleavage mixture was incubated at 4 °C without agitation for 4 days to minimize aggregation. TDP-43_{CTD} was separated from $Thio_6$-His_6-TEV and residual $Thio_6$-His_6-TEV-TDP-43_{CTD} by passing through a second HisPrep 16/10 FF column (Cytiva) and buffer exchanged into a low ionic strength buffer (7 M urea, 20 mM MES, pH 6.0) on a HiPrep 26/10 desalting column (Cytiva). TDP-43_{CTD} was purified by strong cation exchange chromatography on a MonoS 10/100 GL column (Cytiva) and eluted with a NaCl gradient. Purity and identity were assessed by SDS-PAGE and LC-ESI-MS (NHLBI Biochemistry Core). Measured masses are listed below:

WT-TDP-43_{CTD}: 13681.0 Da (calculated: 13680.47 Da)
F283F_{CC}-TDP-43_{CTD}: 13704.9 Da (calculated: 13704.49 Da)
F313F_{CC}-TDP-43_{CTD}: 13705.0 Da (calculated: 13704.49 Da)
W334F_{CC}-TDP-43_{CTD}: 13665.9 Da (calculated: 13665.45 Da)
F367F_{CC}-TDP-43_{CTD}: 13705.1 Da (calculated: 13704.49 Da)
Y374F_{CC}-TDP-43_{CTD}: 13689.0 Da (calculated: 13688.49 Da)
W385F_{CC}-TDP-43_{CTD}: 13665.8 Da (calculated: 13665.45 Da)
F397F_{CC}-TDP-43_{CTD}: 13704.9 Da (calculated: 13704.49 Da)

Purified protein was concentrated to 200 µM in a Millipore Amicon 8400 stirred ultrafiltration cell using a 3 kDa MWCO membrane filter, flash frozen in 200 µL aliquots and stored at −80 °C until use. All buffers were filtered (0.22 µm) and stored at 4 °C.

Protein droplet preparation. Aliquots of TDP-43_{CTD} were thawed at RT and desalted into deionized H_2O on BioSpin6 columns (BioRad). Protein concentration was determined from molar absorptivity at 280 nm based on amino acid content ($\varepsilon_{280\ nm}$ (WT) = 17,990 $M^{-1}cm^{-1}$; $\varepsilon_{280\ nm}$ (FXF_{CC}) = 18,310 $M^{-1}cm^{-1}$; $\varepsilon_{280\ nm}$ (WXF_{CC}) = 12,810 $M^{-1}cm^{-1}$; $\varepsilon_{280\ nm}$ (Y374F_{CC}) = 16,820 $M^{-1}cm^{-1}$) and salt-free samples were prepared at 1.1× working concentration (27.5 µM). LLPS was induced by addition of 10× phosphate buffer (100 mM NaP_i, 2 M NaCl, pH 7.4) and aliquots were immediately transferred to 18 well chambered cover glass with #1.5 high performance cover glass (Cellvis, C18-1.5H). At least three independent aging experiments with multiple time points were performed for all proteins.

Fluorescence imaging. Widefield fluorescence images were acquired on an Evident IX-83 inverted microscope using a 10× objective (Evident UPLFLN10X). Samples were illuminated using an X-Cite 120LED Boost excitation lamp (Excelitas) directed through a FF01-433/24-25 excitation filter (Semrock), a FF435-Di01-25x36 dichroic beamsplitter (Semrock), and fluorescence was collected through a BLP01-458R-25 emission filter (Semrock) and recorded on a Hamamatsu ORCA-Flash4.0 v3 CMOS camera.

Confocal Raman spectroscopy. Samples were imaged in a custom Raman spectral imaging system built around an IX-71 inverted microscope (Evident). Samples were visualized using a 60×/1.3 NA silicon oil immersion objective (Evident UPLSAPO60XS2), and bright field images were acquired using an Infinity3-6URM camera (Teledyne Lumenera). Raman illumination was provided by the 514-nm line of a 35-MAP-431 argon ion laser (Melles Griot) expanded and collimated through a ZBE2A beam expander (ThorLabs) to match the back aperture diameter of the objective. Laser illumination (10 mW) was passed through a LL01-514-25 clean up filter (Semrock) and directed into the objective by a LPD01-514RU-25x36x1.1 dichroic beamsplitter (Semrock). Backscattered light was collected through the same objective and dichroic, filtered through a NF03-514E-25 notch filter (Semrock), and focused through a 220M tube lens adapter (Horiba) into an iHR320 spectrometer (Horiba) with the entrance slit set to 50 μm and equipped with a 1200 mm^{-1} grating. Raman spectra were recorded as the average of 2×10-s accumulations on a Symphony II back-illuminated deep-depleted liquid nitrogen cooled CCD (Horiba) with the gain set to Best Dynamic Range and an ADC setting of 1 MHz. The CCD was binned in the *y*-dimension from pixel 124-132 to achieve confocality. Optimal *z*-plane for data collection was determined from a *z*-scan using the maximum alkyne peak intensity (~ 2 μm above the coverglass). Spectra of protected F_{CC} (N-acetyl-4-ethynyl-L-phenylalanine methyl ester) in neat solvents were acquired in the same manner, but with a laser power of 124 mW using a 60×/1.2 NA water immersion objective (Evident UPLSAPO60XW), and with spectra recorded as the average of 512×0.125-s accumulations.

Raman data analysis. Raman data analysis was performed in LabSpec 6.7.1.10 (Horiba). The Raman shift was calibrated daily by performing a 2nd degree polynomial fit of a neat cyclohexane spectrum to published peak frequencies.[4] Background spectra collected outside droplets within each sample were smoothed using a 2nd degree polynomial with a 20-point window and subtracted from each droplet spectrum. A 5th degree polynomial baseline correction was applied to each spectrum, and the spectrum was normalized to either the area of the C–H deformation band calculated from 1396 to 1478 cm^{-1} or the alkyne stretching band calculated from 2064 to 2140 cm^{-1}. Difference spectra were calculated in Excel (Microsoft) by averaging the spectra of all nascent droplets for each mutant and subtracting this spectrum from individual spectra of aged droplets or fibrils. Nascent droplet spectra were collected from freshly prepared samples within 3 h of aged droplets and fibrils in order to eliminate slight day-to-day Raman shift changes. Figure plots were made in Igor Pro 9.0.5.1. Peak positions and widths were determined by fitting to a linear combination of two Gaussian functions in Igor Pro 9.0.5.1 and extracting values from the resulting function.

Transmission Electron Microscopy. TDP-43_{CTD} droplets were prepared as described and deposited on Formvar/carbon supported 400 mesh copper grids (Electron Microscopy Sciences). After overnight incubation in a humidity chamber at room temperature, the excess liquid was wicked away with filter paper. The grid was washed with a drop of water and stained with 1% uranyl acetate for 1 min. Grids were imaged on a JEOL EM-1200 EXII electron microscope (accelerating voltage 80 keV) equipped with an AMT XR-60 digital camera (NHLBI EM Core Facility).

(1) Shuster, S. O.; Lee, J. C. Watching Liquid Droplets of TDP-43(CTD) Age by Raman Spectroscopy. *J. Biol. Chem.* **2022**, *298* (2), 101528.
(2) Miyake-Stoner, S. J.; Miller, A. M.; Hammill, J. T.; Peeler, J. C.; Hess, K. R.; Mehl, R. A.; Brewer, S. H. Probing Protein Folding Using Site-Specifically Encoded Unnatural Amino Acids as FRET Donors With Tryptophan. *Biochemistry* **2009**, *48* (25), 5953–5962.
(3) Hammill, J. T.; Miyake-Stoner, S.; Hazen, J. L.; Jackson, J. C.; Mehl, R. A. Preparation of site-specifically labeled fluorinated proteins for 19F-NMR structural characterization. *Nat. Protoc.* **2007**, *2* (10), 2601–2607.
(4) International, A. *Standard Guide for Raman Shift Standards for Spectrometer Calibration*; E1840; 2022. DOI: 10.1520/e1840-96r22.
(5) Romei, M. G.; von Krusenstiern, E. V.; Ridings, S. T.; King, R. N.; Fortier, J. C.; McKeon, C. A.; Nichols, K. M.; Charkoudian, L. K.; Londergan, C. H. Frequency Changes in Terminal Alkynes Provide Strong, Sensitive, and Solvatochromic Raman Probes of Biochemical Environments. *J. Phys. Chem. B* **2023**, *127* (1), 85–94.
(6) Laurence, C.; Legros, J.; Vuluga, D. A Collection of Dispersion Induction DI, Electrostatic ES, and Hydrogen Bonding alpha(1) and beta(1) Parameters for 380 Solvents and What They Say on Solvent Effects on Rates, Equilibria, and Spectra. *J. Org. Chem.* **2024**, *89* (13), 9521–9542.

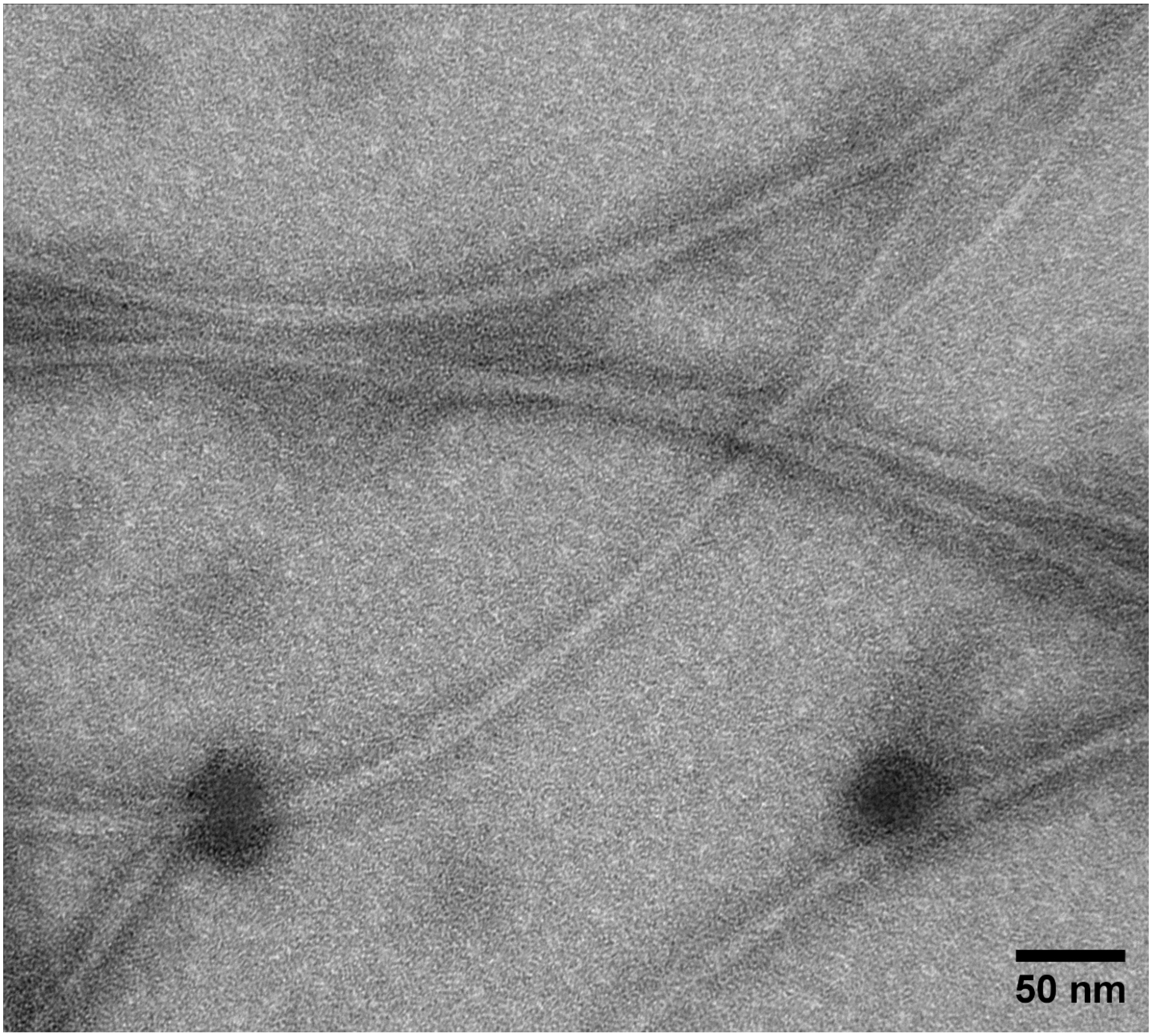

Figure S1. Transmission electron micrograph of TDP-43$_{CTD}$ fibrils.

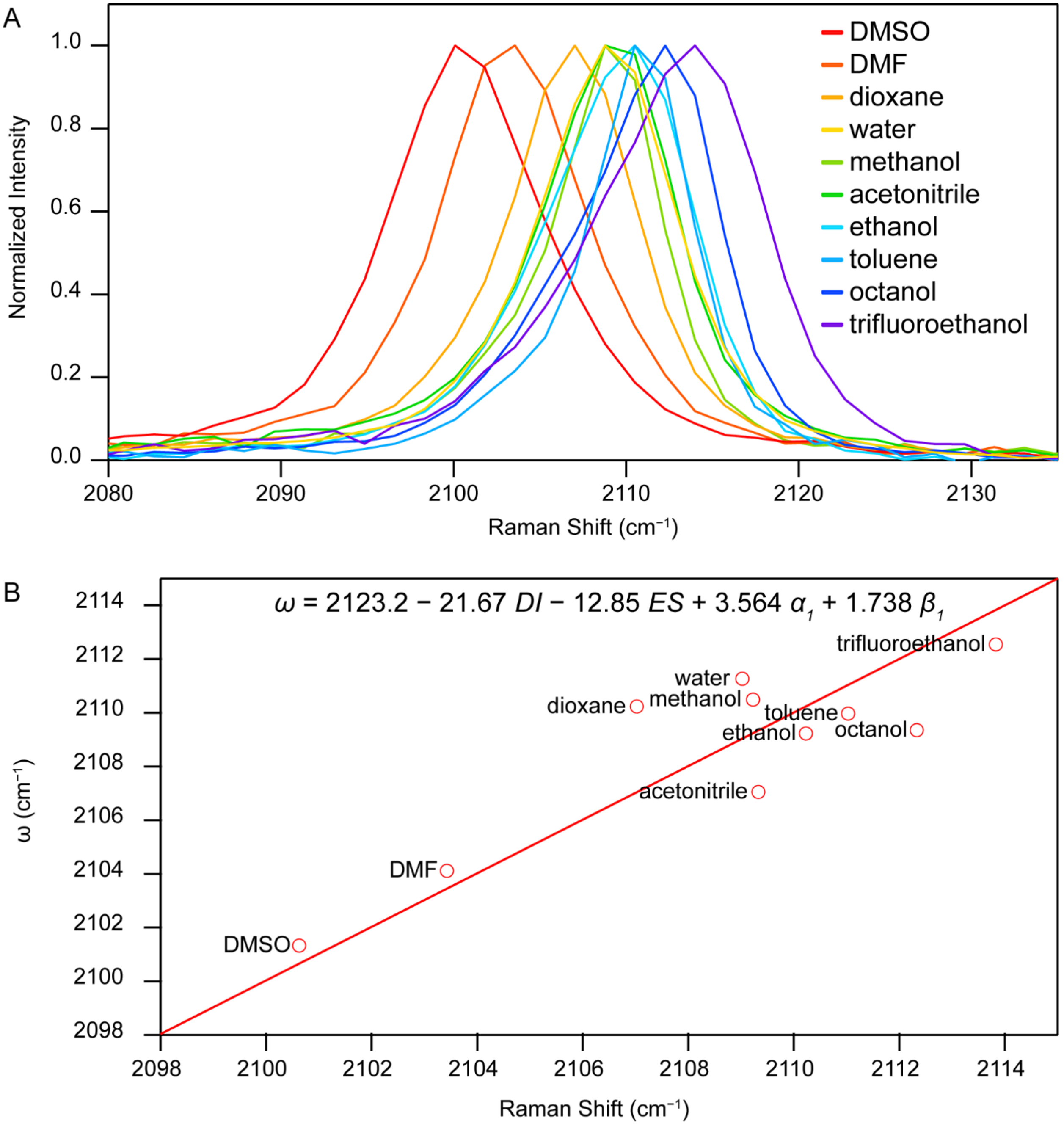

Figure S2. (A) C≡C region (normalized to the intensity maximum) of a protected variant of F_{CC}, N-acetyl-4-ethynyl-L-phenylalanine methyl ester in different solvents. (B) C≡C peak frequency of N-acetyl-4-ethynyl-L-phenylalanine methyl ester *versus* the predicted values (ω) from fitting to solvent parameters following the analysis of Romei *et al.*.[5] The equation determined by linear regression for the relationship between ω and published solvent dispersion-induction (*DI*), electrostatic (*ES*), H-bond donating (α_1), and H-bond accepting (β_1) parameters[6] is shown. The diagonal (red line) is shown as a visual guide.

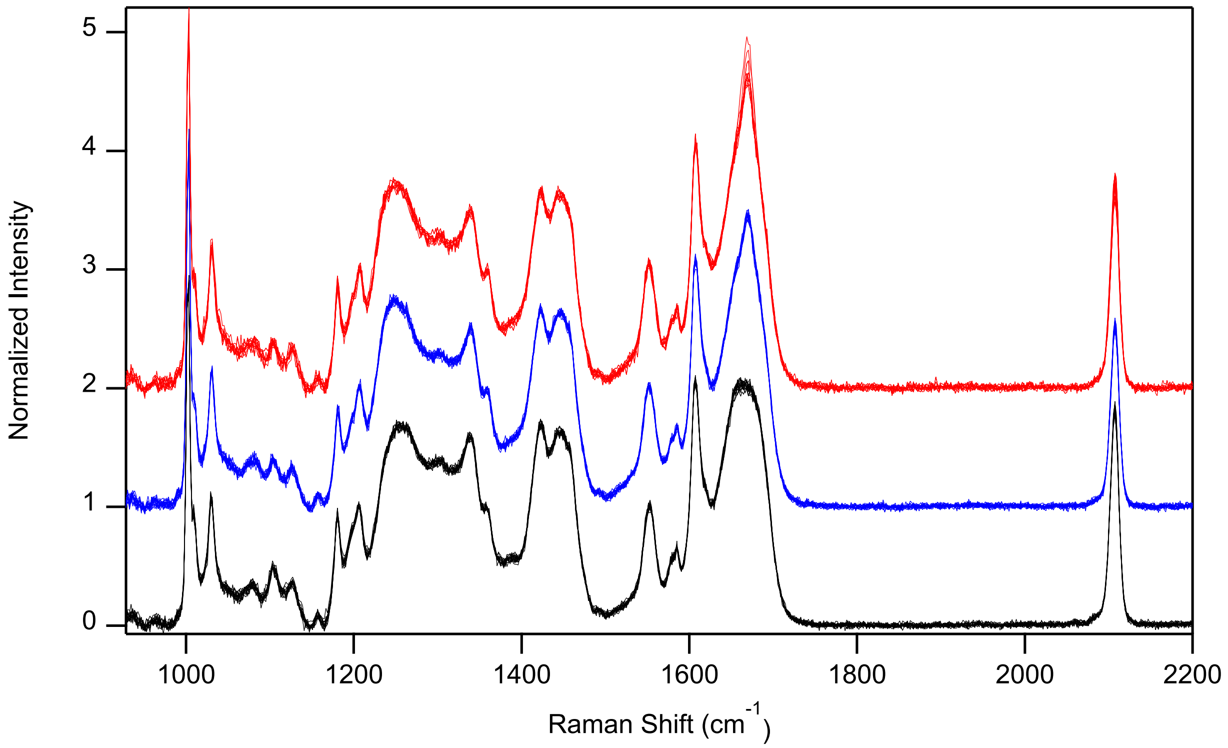

Figure S3. Raman spectra of F283F$_{CC}$-TDP-43$_{CTD}$ in the nascent droplet (black, n = 16), aged droplet (blue, n = 16) and fibrillar (red, n = 16) states. All spectra are normalized to the area of the C–H deformation band from 1396 to 1478 cm^{-1}.

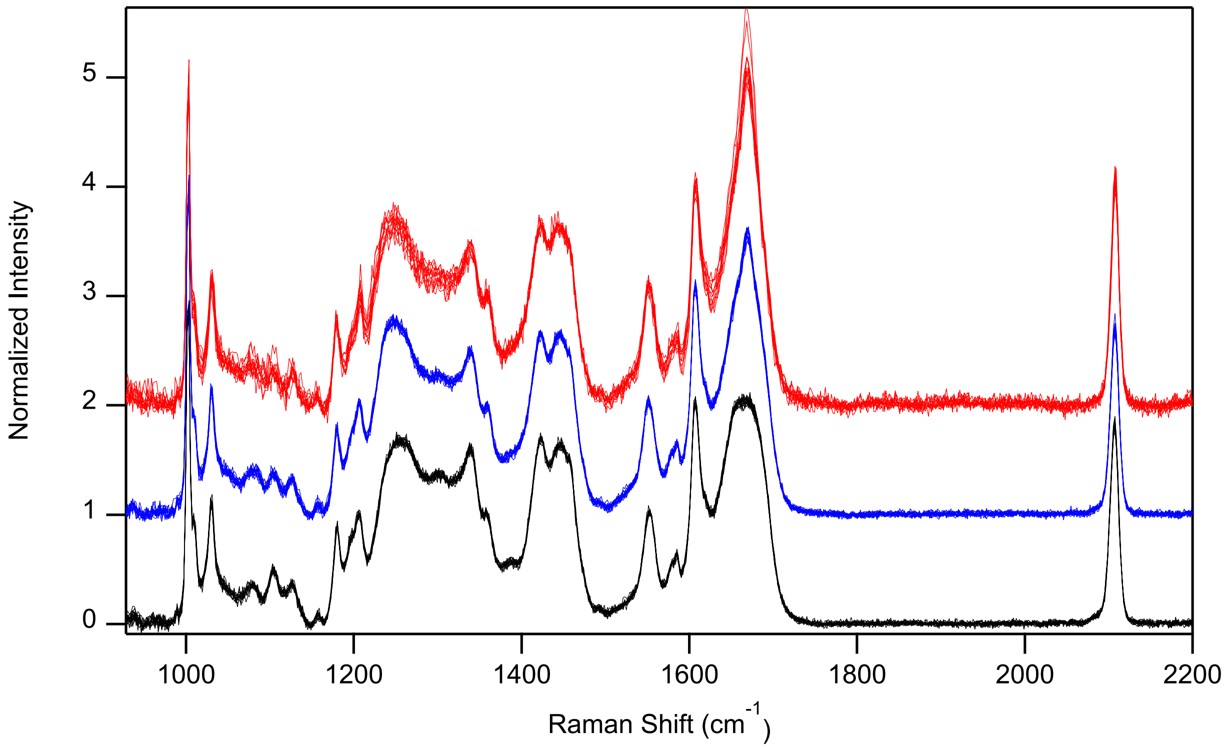

Figure S4. Raman spectra of F313F$_{CC}$-TDP-43$_{CTD}$ in the nascent droplet (black, n = 16), aged droplet (blue, n = 16) and fibrillar (red, n = 13) states. All spectra are normalized to the area of the C–H deformation band from 1396 to 1478 cm^{-1}.

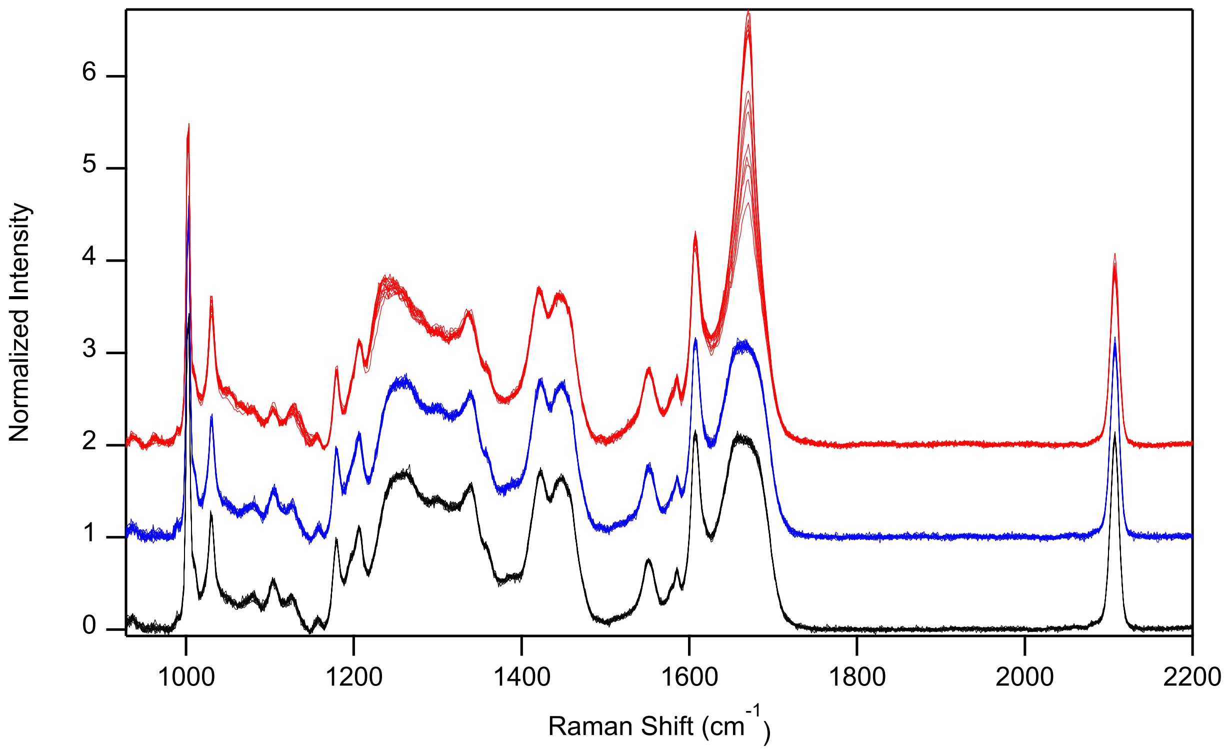

Figure S5. Raman spectra of W334F$_{CC}$-TDP-43$_{CTD}$ in the nascent droplet (black, *n* = 16), aged droplet (blue, *n* = 16) and fibrillar (red, *n* = 16) states. All spectra are normalized to the area of the C–H deformation band from 1396 to 1478 cm^{-1}.

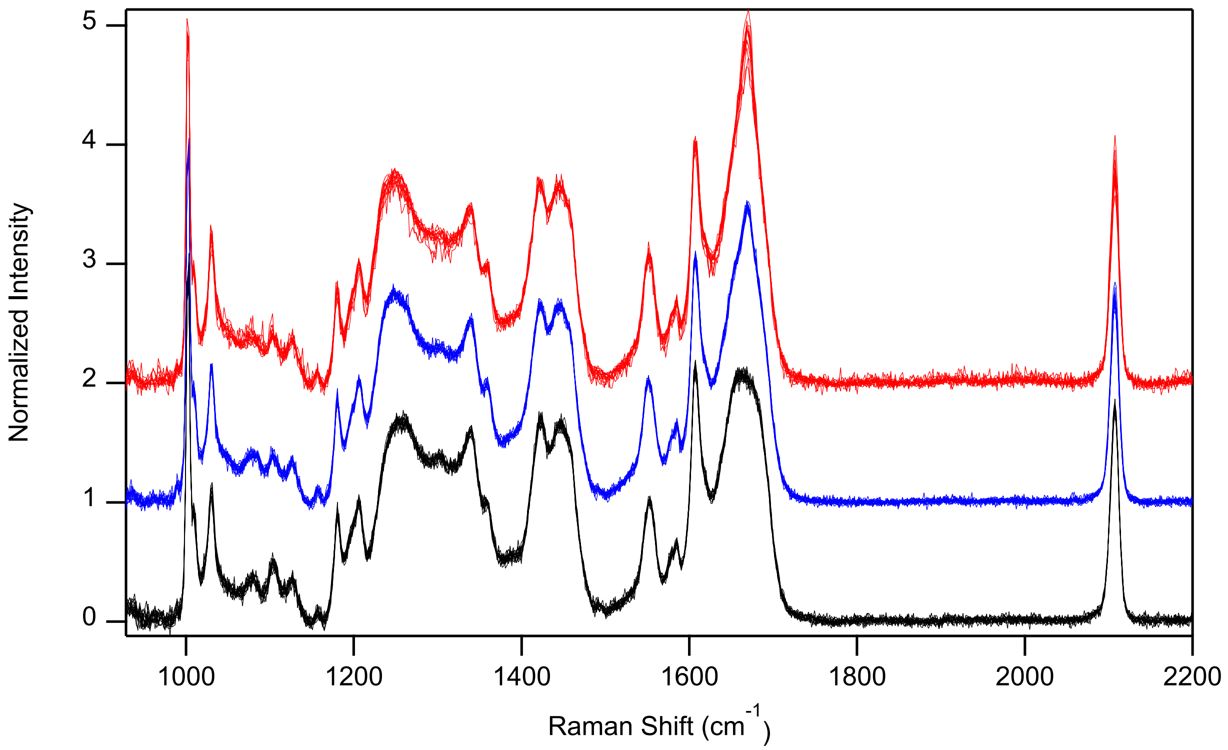

Figure S6. Raman spectra of F367F$_{CC}$-TDP-43$_{CTD}$ in the nascent droplet (black, *n* = 16), aged droplet (blue, *n* = 16) and fibrillar (red, *n* = 14) states. All spectra are normalized to the area of the C–H deformation band from 1396 to 1478 cm^{-1}.

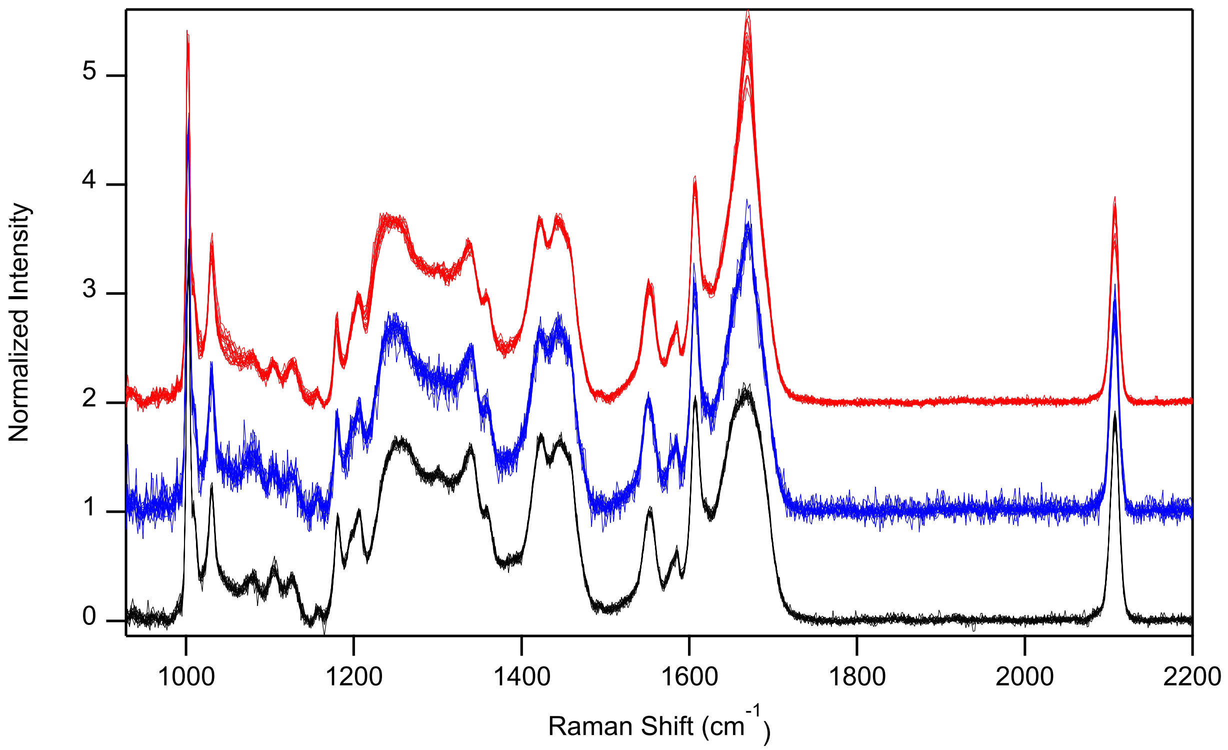

Figure S7. Raman spectra of Y374F$_{CC}$-TDP-43$_{CTD}$ in the nascent droplet (black, *n* = 16), aged droplet (blue, *n* = 15) and fibrillar (red, *n* = 17) states. All spectra are normalized to the area of the C–H deformation band from 1396 to 1478 cm^{-1}.

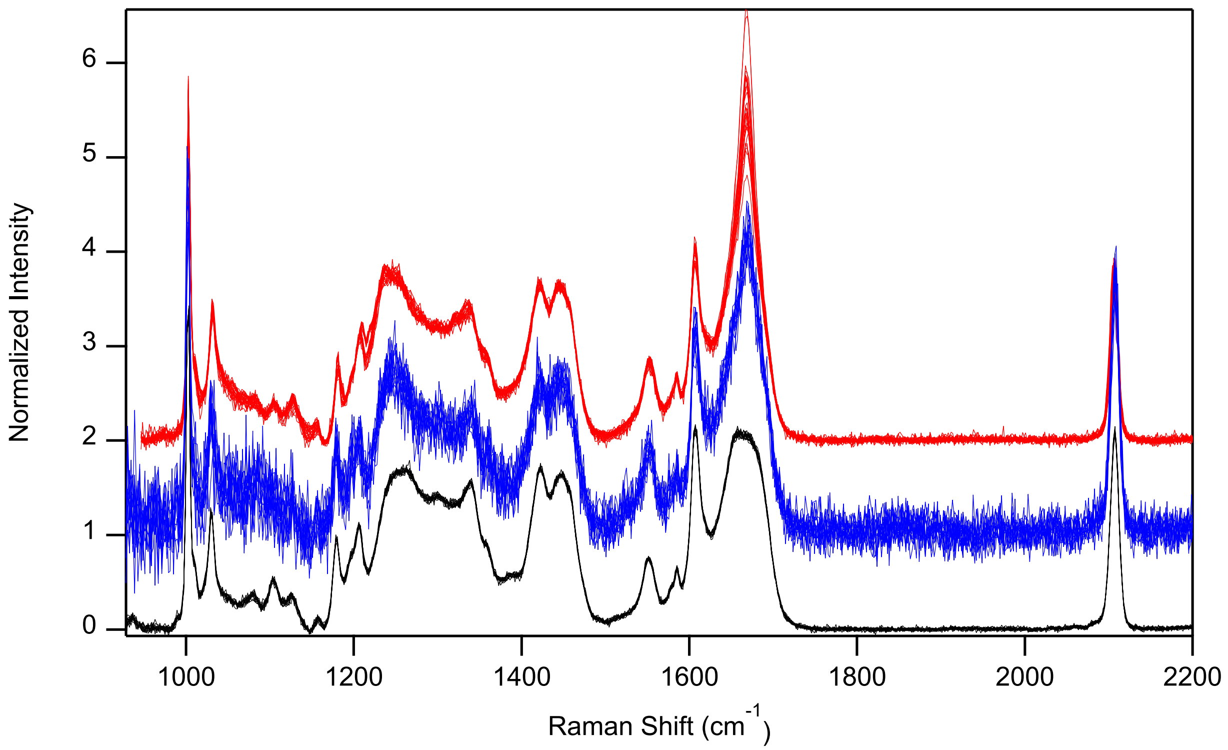

Figure S8. Raman spectra of W385F$_{CC}$-TDP-43$_{CTD}$ in the nascent droplet (black, *n* = 16), aged droplet (blue, *n* = 14) and fibrillar (red, *n* = 30) states. All spectra are normalized to the area of the C–H deformation band from 1396 to 1478 cm^{-1}.

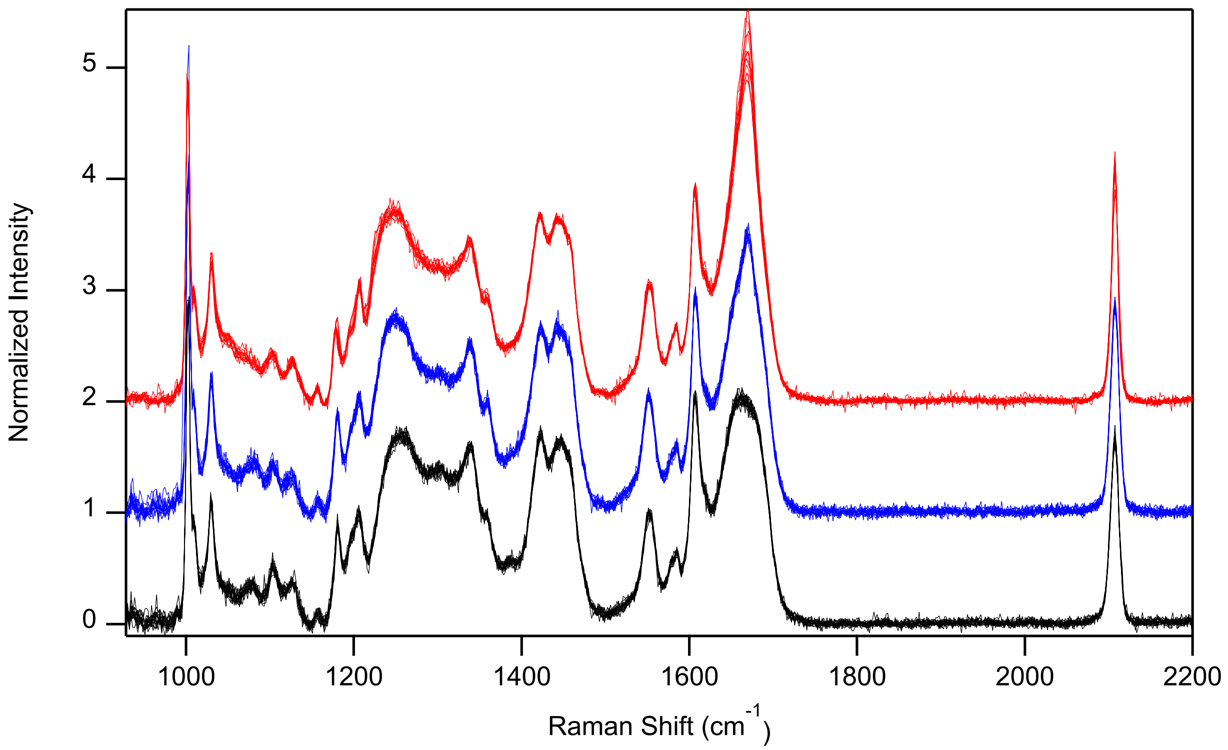

Figure S9. Raman spectra of F397F$_{CC}$-TDP-43$_{CTD}$ in the nascent droplet (black, *n* = 17), aged droplet (blue, *n* = 15) and fibrillar (red, *n* = 15) states. All spectra are normalized to the area of the C–H deformation band from 1396 to 1478 cm^{-1}.

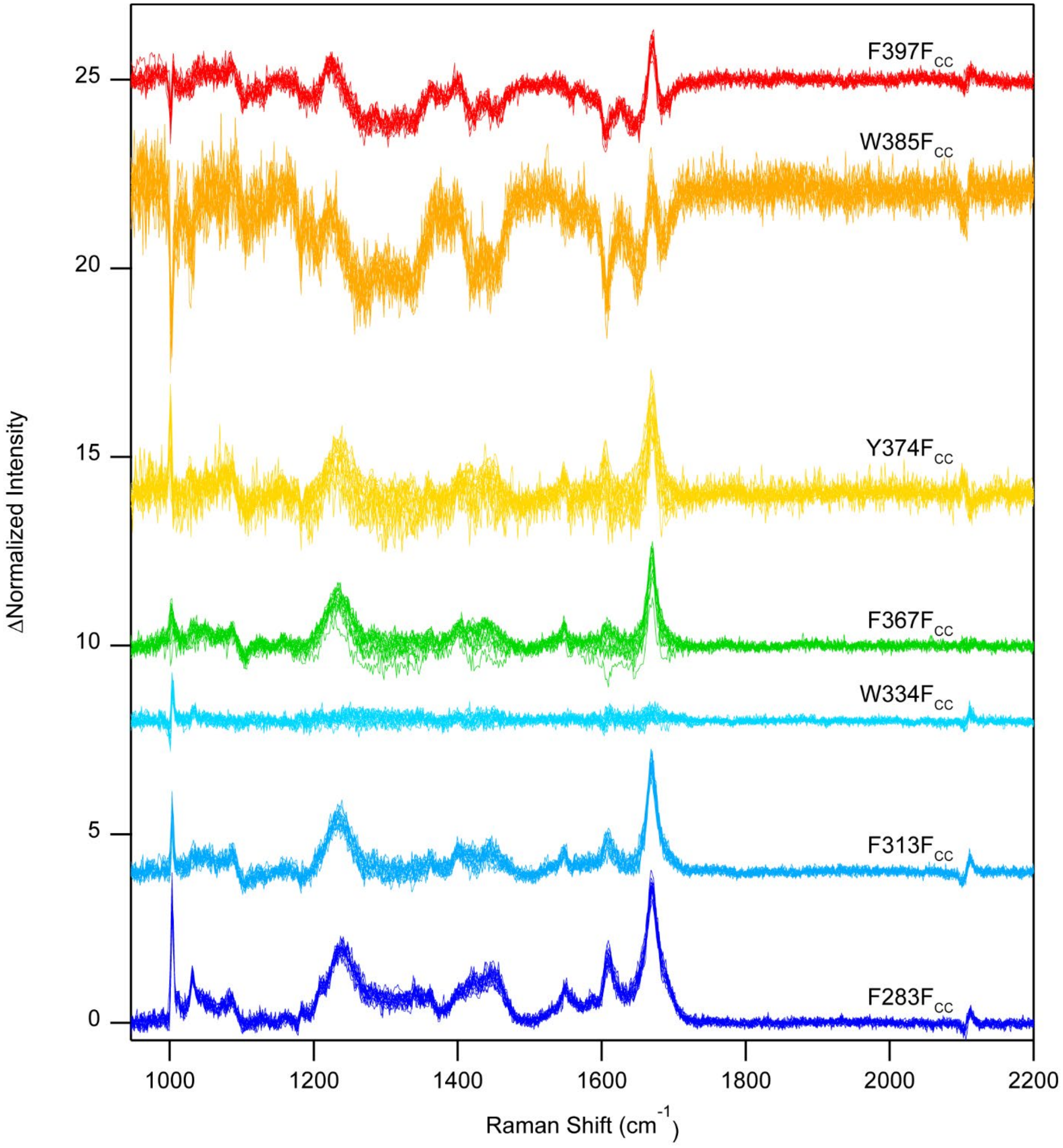

Figure S10. Full spectral range of spectra shown in **Fig. 2L**. Raman difference spectra of aged F_{CC}-TDP-43$_{CTD}$ droplets minus the average spectrum of their respective nascent droplets. All spectra are normalized to the area of the C≡C stretching band from 2064 to 2140 cm^{-1}.

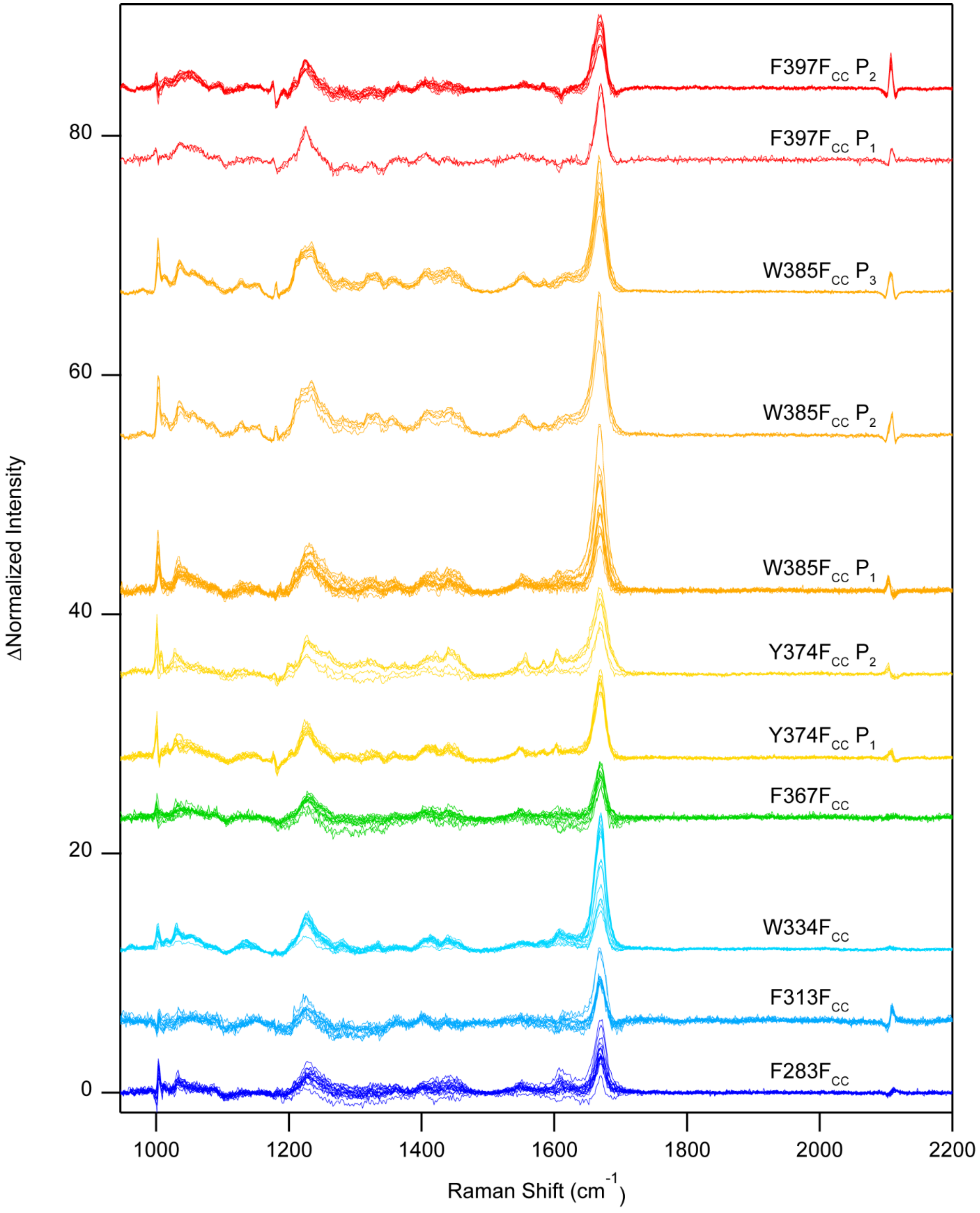

Figure S11. Full spectral range of spectra shown in **Fig. 2M**. Raman difference spectra of F$_{CC}$-TDP-43$_{CTD}$ fibrils minus the average spectrum of their respective nascent droplets. Spectrally distinct polymorphs (P) are grouped as P$_{1–3}$. All spectra are normalized to the area of the C≡C stretching band from 2064 to 2140 cm^{-1}.

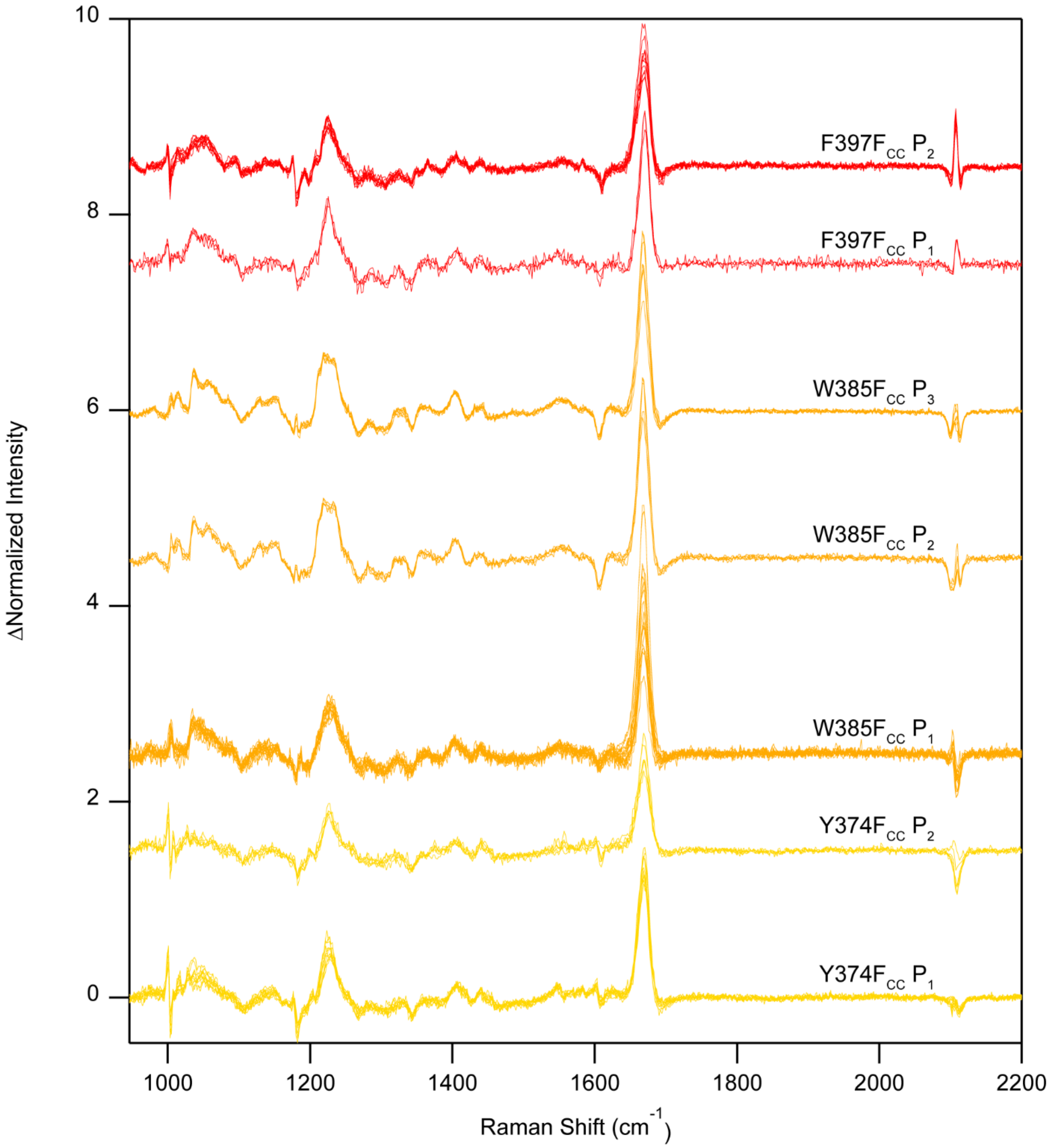

Figure S12. Full spectral range of spectra shown in **Fig. 2N**. Raman difference spectra of spectrally distinct F_{CC}-TDP-43_{CTD} fibril polymorphs ($P_{1\text{-}3}$) minus the average spectrum of their respective nascent droplets. All spectra are normalized to the area of the C–H deformation band from 1396 to 1478 cm^{-1}.
